# Less is more: Biased loss of CpG dinucleotides strengthens antiviral immunity

**DOI:** 10.1371/journal.pbio.3001353

**Published:** 2021-09-08

**Authors:** Daniel Sauter, Frank Kirchhoff

**Affiliations:** 1 Institute for Medical Virology and Epidemiology of Viral Diseases, University Hospital Tübingen, Tübingen, Germany; 2 Institute of Molecular Virology, Ulm University Medical Center, Ulm, Germany

## Abstract

Viruses may not only affect our daily lives but also shape our genome evolution. A recent study shows that the zinc-finger antiviral protein (ZAP) drives CpG suppression in a biased manner. Genes involved in the defense against viral invaders are particularly CpG suppressed to avoid self-targeting and to promote an effective immune response.

Upon viral infection, our innate immune system needs to initiate efficient defense mechanisms attacking the pathogen without markedly harming the host. Distinguishing self from nonself represents a formidable challenge since all viral components are generated by the host cell. Although viruses and their hosts use the same cellular machineries, viral and cellular RNAs show differences in codon usage, nucleotide composition, and the presence of specific secondary structures. These characteristics are exploited by antiviral host factors to preferentially target viral RNA, while sparing cellular transcripts. However, the establishment of an antiviral mechanism initiates an arms race: Viruses mimic the features of their hosts to evade restriction, while the host will evolve toward more pronounced differences to maintain discrimination between self and nonself. Over millions of years of coevolution, this interplay had a major impact on the composition of host genomes.

A prime example for this phenomenon is the marked suppression of CpG dinucleotides in mammalian genomes [1]. For example, CpGs are more than 4-fold less frequent in the human genome than expected. Many viruses mimic this host feature [2]. Just recently, the zinc-finger antiviral protein (ZAP) has been identified as a driving force behind this suppression of CpG dinucleotides [3]. It has been shown that ZAP specifically binds to CpG dinucleotides in HIV-1 mRNAs to target them for degradation. ZAP has an ancient origin [4] and is capable of restricting a large variety of viral pathogens including retro-, alpha-, filo-, hepadna-, picorna-, toga-, herpes-, corona-, and flaviviruses [5]. Thus, ZAP may have driven CpG suppression in many viruses and their respective host species. Notably, ZAP itself lacks nuclease activity and requires the RNase KHNYN to degrade viral RNAs [6].

Effective distinction between self and nonself is especially important in the presence of a viral pathogen, i.e., when host defense mechanisms are induced and highly active. Sensing of viral pathogens triggers the production of interferons (IFNs), which induce the expression of hundreds of interferon-stimulated genes (ISGs) and establish an antiviral state of the cell. Since ZAP is also an ISG [7], the degradation of CpG-rich RNAs increases during the innate antiviral immune response. It would obviously be counterproductive if ZAP suppressed the expression of other antiviral defense factors. Now, Shaw and colleagues revealed how the host avoids detrimental self-targeting of ISG transcripts [8]. By performing comprehensive computational analyses, they demonstrate that on average, ISGs show particularly strong CpG suppression. Similarly, the study revealed that IFN genes are frequently extremely suppressed in CpGs, although this was not observed in all species. In contrast, interferon-repressed genes (IRGs) were frequently relatively CpG rich. Shaw and colleagues even identified CpG content as the most definitive feature distinguishing ISGs from IRGs. These findings suggest that ZAP has a major impact on the composition of IFN-modulated host transcripts, i.e., the interferome.

Shaw and colleagues provide evidence that following IFN treatment, IRGs are frequently suppressed by ZAP, while ISGs are hardly affected. Thus, particularly effective CpG suppression promotes the maintenance of effective IFN responses and antiviral gene expression upon ZAP induction (Fig 1). While the study shows that ZAP accounts for the repression of some IRGs, it remains to be determined whether this repression just represents a tolerable side effect of ZAP or whether their silencing helps to suppress viral replication. It will be a challenging task to clarify this because suppressive effects on IRGs are usually modest (<2-fold), while ISGs may be up-regulated by several orders of magnitude. Notably, ZAP depletion was not only associated with increased expression of relatively CpG-rich genes but also down-regulation of a significant number of genes. The authors speculate that this might be due to increased expression of genes suppressing transcription. However, the underlying mechanisms and the significance of ZAP-mediated effects on cellular gene expression for the antiviral immune response and beyond warrant further study.

**Fig 1:**
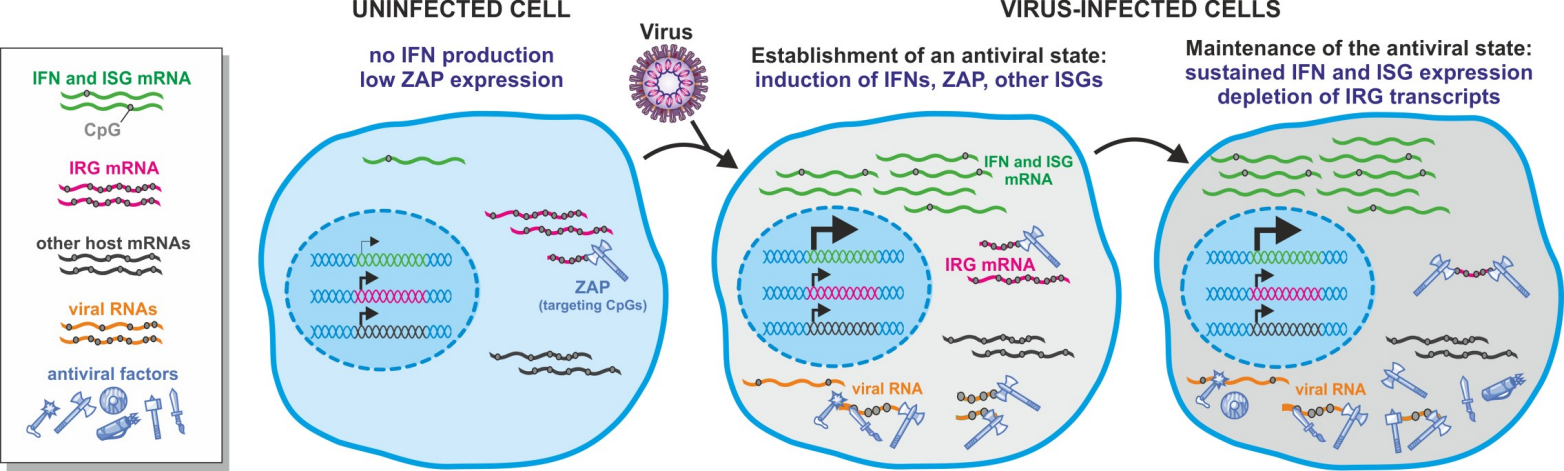
Biased CpG suppression in cellular genes to maintain efficient innate antiviral immune responses in the presence of the antiviral factor ZAP. Uninfected cells (left) express a large variety of mRNAs showing significant but varying magnitudes of CpG suppression. Virus infection (middle) triggers the IFN response associated with strong induction of IFNs and numerous ISGs, expressing a large array of structurally and functionally diverse antiviral factors. One of them is ZAP, which mediates degradation of CpG-rich viral as well as cellular mRNAs. Particularly strong CpG suppression allows mRNAs encoding IFNs and antiviral factors to evade degradation by ZAP and, thus, to maintain an effective antiviral state (right). Some cellular genes are suppressed by the IFN response, and this effect is partly dependent on ZAP expression. Whether IRGs might otherwise promote viral replication directly or by attenuating the antiviral immune response remains to be determined. Color coding of viral and cellular RNA species and symbols is explained on the left. IFN, interferon; IRG, interferon-repressed gene; ISG, interferon-stimulated gene; ZAP, zinc-finger antiviral protein.

Shaw and colleagues analyzed the CpG content of ISGs and IRGs from a variety of species covering over 300 million years of evolution. They provide compelling evidence that CpGs are more rapidly purged from ISGs than from IRGs and other cellular genes. Overall, increased CpG suppression of highly IFN-responsive ISGs was evolutionarily conserved. However, the authors also identified marked differences between different species. For example, canine ISGs contained 3-fold higher levels of CpGs than IRGs, while only marginal differences were found in equines. In addition, IFN genes showed extremely low CpG content in most species but not in horses and were even relatively CpG rich in chickens. Again, it will be of significant interest to determine whether this might be merely due to misannotated sequences, differences in ZAP activity, or evasion from ZAP restriction independently of CpG depletion.

The results of Shaw and colleagues agree with previous evidence that CpG numbers are an important, but are not the only, determinant of ZAP sensitivity. For example, the average CpG content of genes suppressed upon IFN stimulation was remarkably similar in the presence and absence of ZAP. It has been shown that RNA structure and/or the number of CpGs at specific locations of viral RNA rather than overall CpG frequency may determine ZAP sensitivity [9–11]. Notably, ZAP targeting is a highly plausible but not the only possible reason for CpG suppression in highly inducible genes. For example, DNA methylation at CpG dinucleotides is associated with silencing of cellular gene expression. Thus, the need for efficient induction may have contributed to the loss of CpGs in immune-associated genes.

The study by Shaw and colleagues [8] adds to a growing body of evidence that human evolution is under numerous constraints and that our genome has been shaped to a large extent by viruses. In order to maintain effective innate antiviral defense mechanisms, ZAP not only drives CpG suppression but also resulted in a significant bias in the composition of the vertebrate interferome to avoid self-targeting during the antiviral immune response. ZAP is not the only cellular factor targeting viral RNAs. Thus, further studies on how the need to distinguish self from nonself has influenced (and is continuing to influence) the composition of genes in general and may select for bias in specific genes due to differences in evolutionary pressures will be of significant interest.

## Abbreviations

IFN: interferon
IRG: interferon-repressed gene
ISG: interferon-stimulated gene
ZAP: zinc-finger antiviral protein
